# Why I like Chest Physicians

**Published:** 1991-09

**Authors:** J. L. Burton

**Affiliations:** Consultant Dermatologist, Bristol Royal Infirmary


### Why I like chest physicians

**J. L. Burton, BSc, MD, FRCP**
Consultant Dermatologist, Bristol Royal Infirmary

We all have our reasons, which may in some cases be weird and in others wonderful, but in my case its simply because the first two chest physicians I ever met, both gave me such excellent advice.

It was one day in the Upper Sixth at the local Grammar School that the fourth or maybe the fifth uproarious joke of the day made me laugh so much that I had a coughing fit, and what I coughed, stained my handerchief bright red. I'd heard of Keats and what killed him, and I was worried. In those days 'conspicuous consumption' was even less desirable than it is now.

The G.P. was disarmingly pleasant and relaxed.

"Have you been feeling tired lately?" Well as a matter of fact I used to finish 3rd or 4th in the cross-country races, but lately I'd been training harder and harder and finishing 20th or even 30th, but that's life.

"Any tuberculosis in the family at all?" Well my uncle had died of it some years ago — not that this was unusual in the Derbyshire mining-town where I lived. Most of the male adult population had blue coal-dust tattoos on their forehead, and they nearly all had anthracosis, bronchitis, pulmonary T.B. or all three.

"Hmm, I think we'd better have a chest X-ray, just to be on the safe side".

This was duly reported as showing "a shadow" and the G.P. explained I was likely to be in a Sanatorium for "quite a long time". There was no discussion of the prognosis, and since I well knew that tuberculosis killed people I walked sadly around the local fields for the last time saying goodbye to well-loved landscapes. Seriously preparing for death in this way during adolescence has a lot to recommend it.... it puts the rest of one's life in perspective.

Thus it was that in January 1957 I was admitted for an indeterminate period to the Derwent Sanatorium. It was not a wooden chalet on a pine-scented hill-side in Switzerland, as the books had led me to believe. It was red-brick and sooty, on the outskirts of Derby. And there I received an education in the ways of life and more importantly, death.

I was welcomed to the ward by the three old lags who seemed to act as a joint but benevolent Godfather, at least as far as card-schools and betting-slips were concerned. They shared approximately one lung between them, and were remarkably similar. They were all very thin, they had blue lips, they walked slowly (but only if they had to) and when they talked, they gasped. Between gasps they spat copious amounts of white fluid into pint sputum mugs which they carried everywhere. They'd each been in and out of sanatoria for about 30 years, and they carefully explained to me that whilst a young man who behaved sensibly might expect eventually to be discharged from a T.B sanatorium, he could not expect to stay discharged forever. Sooner or later a patient would always come back, and though he might go home again, even several times, eventually the little bacillus would always win.

Jim was a case in point. I'd been given a side-room because I had to work for my A level exams, and Jim was in the room next door. He was too ill to talk, but I used to hear him gasping and moaning in the night. My vulturine cronies estimated he had about a week to go, and after 9 days, he went. Their predictions about the death, discharge and readmittance of other patients in the ward were also fairly accurate, and it took some time before I realized that their prognostic expertise was based on a vast experience of advanced cases from the pre-treatment era. It was several months before I learnt that some patients were actually curable.

I can't recall seeing a chest physician at that stage. Authority took the form of a male Charge-Nurse and a male Staff-Nurse, both of whom were ex-military gentleman with bristling moustaches. They had, so far as I could see, two main functions. The first was to stand no nonsense when, thrice daily, we swallowed the absolutely revolting PAS (para-amino salicylic acid) medicine. They would then administer the very painful intra-gluteal streptomycin injections, ignoring the colourful language this provoked, unless it was of an unduly personal nature. The isoniazid pills were no problem, especially as they were accompanied by vitamin B6 tablets which were reputed to possess aphrodisiac properties. Patients approaching 'home leave' tended to hoard them.

The second function of our moustachioed guardian angels was to ensure that we all lay to attention during the two compulsory 'rest-hours' each day, and didn't go to sleep or read books. They explained that we must not sleep during the day, as otherwise we wouldn't sleep at night and would disturb the night staff. This was true, particularly since the night staff were neither military nor male. Conversely we musn't read books during the 'rest-hour', as they would be too 'stimulating'. Judging by the grimy well-thumbed state of what the other inmates read, that might well also have been true.

At regular intervals we were sent for a chest X-ray and weighed, the dual aims of treatment being to decrease the 'shadow' and increase the substance. In my case the substance increased by about a stone and a half. The Charge-Nurse would tersely convey the result of the X-ray. "A slight improvement, do as you're told and you'll be alright". It seemed an impertinence to ask whether, let alone when, I might eventually go home.

After five months or so my chest X-ray was pronounced clear and I was examined by the Registrar, whom I shall call Dr. Sullivan. He was a large, cheerful Irishman who'd had problems with the MRCP examination. After he'd examined my chest he said I was fine, and I could expect to go home very soon. But I could sense that something was troubling him. At length the cause of his concern became apparent.

"Dey tell me ye've passed yer A levels wid floyin' cullurs?"

"Yessir" (proud smile).

"And ye've bin offered a pleyce at Manchester Medical School?"

"Yessir" (broader smile).

"So ye'll be all set to be a proper medical stoodent from next October den?"

"Yessir" (wide grin).

Pregnant pause, followed by a slow, sad shake of the head "Ye poor bastard".

I was totally nonplussed by this. Hadn't I just read 'Doctor in the House'?

"Well, oi'll wish ye more joy of it than oi've had of it meself so far. Oi'm a Dublin man meself, but wherever ye cum frum it seems ter me ye have ter struggle. Its not all beer and skittles oi can tell ye; its bluddy hard wurk, and the sooner ye realize that, the better. Anyway good luck to ye, laddie".

And with that he clapped me on the shoulder and was gone. Off to Australia the following week, so the Staff-Nurse told me.

On reflection this sobering advice was not without its merit. Medicine **isn't** all beer and skittles, it mostly **is** bloody hard work, and the advance warning did me no harm at all.

The second piece of useful advice came shortly afterwards, from the consultant, whom I shall call Dr. Jones. He was a small wizened man, with a stern expression. "There's good news and there's bad. The good news is that your tuberculosis is now totally inactive and is likely to remain so. The bad news is that I'm not willing to authorize you to start the hurly-burly of medical school life so soon as next October. I think you should have a year's convalescence. If I let you go to University too soon and you start having too many late nights and your T.B. breaks down again, you'll be back in and out of here for the rest of your life, and that'll be the end of your career. Its not a risk worth taking. Any chance of you spending some time travelling abroad for 6 months or so?''

About as much chance as winning the football pools actually, since the one would depend on the other.

''No, I rather thought not: Well, what you should do is go to your local Library and spend the next year reading as many great works of literature as you possibly can. It doesn't really matter what you read, as long as its interesting and it makes you think''.

That sounded sensible to me, and that's more or less what I did. I lived a hermit-like existence at home, bought a pair of chest expanders, turned a stone and a half of fat into muscle, and twice a week I went to the local Library and brought home a bagful of books, most of which I read.

Those were the heady days before cuts in local Government expenditure, when even small-town Libraries were well-stocked with proper books.

The practical benefits of this year-long literary immersion did not become apparent to me until my first term at University. It is possible that the public-school-educated Oxbridge medics had a firm grasp of the classics of world literature, but this was certainly not the case with the typical Manchester medic. of 1958. It was relatively unusual in those days to find a medic. with a firm grasp of anything other than a bottle of beer and a member of the opposite sex.

There were, on the other hand, large numbers of female Arts students who had come from genteel homes in the stockbroker belt of Cheshire, but who for one reason or another had failed to make it to a Sloane-type University. These young ladies had for the most part led rather sheltered lives in single-sex boarding schools, until they were eventually let loose in Manchester to learn about life and, it must be admitted, in some cases, not death, but procreation. Such young ladies, having already met a number of what later became known as Hooray Henrys during their school holidays, were often anxious to enlarge their experience of life by meeting muscular young men with broad Northern accents and a refreshing approach. Most of these young ladies had been reading 'Lady Chatterley's Lover' and 'Room at the Top' and were naturally interested in social nuances.

I would occasionally be invited round for coffee by such young persons, but after a desultory chat they would quickly realize I was rather shy, I'd been nowhere and done nothing, and wouldn't know a canapé if it bit me. Visibly disappointed, they would suddenly remember an urgent essay they simply must write that very evening.

''Oh aye'' I'd say, ''What's it on?'' It hard to be sure after 30 years that those were the exact words I used, but certainly the vowels were very flat, and I too had read 'Lady Chatterley's Lover' and greatly admired Mellors the gamekeeper.

By now however the young lady would be well and truly switched off, and would say coolly, as she opened the door and showed me the stairs, ''Well, if you must know, its entitled ''The imagery of death in the works of John Donne''.

''Oh, that's a **fascinating** subject'' I'd say, with genuine enthusiasm, ''Clay Hunt deals with that very well in his 'Essays in Literary Analysis'.

This generally caused some slight hesitation. ''So you've heard of Donne then?''

''Well, who hasn't? Clerical gent, died in 1631, one-time Dean of St. Paul's. If I were you I'd start with 'The Good-Morrow'. The last verse of that poem would be very relevant, you see, where he alludes to the medieval belief that death comes to earthly creatures as a result of the imperfect and inharmonious combination of their elements, as opposed to the harmony of elements seen in spiritual entities, including love of course....

'My face in thine eyes, thine in mine appears,
And true plain hearts do in the faces rest;
Where can we find two better hemispheres
Without sharp north, without declining west?
Whatever dies was not mixed equally;
If our two loves be one, or thou and I
Love so alike that none do slacken, none can die''.

The effect of this declamation at the top of the stairs was often very interesting, and I'm still not sure I fully understand it. Their eyes would go misty, and they'd say something like ''Why don't you come back in for another cup of coffee. Sit on the couch there and I'll just lock the door in case Sandra comes back early. I don't think she will though, she was round at her boyfriend's place till 3 a.m. yesterday. Now what was that last bit again, about 'If our two loves be one'?''

Thank you Dr. Jones, for your sound advice, and if only I'd taken less notice of Dr. Sullivan I'd have had even more fun.

